# The Role of the Functionalization of Biomedical Fabrics on Their Ability to Adsorb and Release Drugs

**DOI:** 10.3390/molecules30030552

**Published:** 2025-01-25

**Authors:** Lucio Melone

**Affiliations:** 1 Department of Chemistry, Materials and Chemical Engineering “G. Natta”, Politecnico di Milano, Via Mancinelli 7, 20131 Milano, Italy; lucio.melone@polimi.it or lucio.melone@uniecampus.it; 2 Department of Theoretical and Applied Sciences, eCampus University, Via Isimbardi 10, 22060 Novedrate, Italy

**Keywords:** cellulose, drug delivery, antibiotics, anti-inflammatory, ibuprofen, amoxicillin, functionalization, cotton fabrics, adsorption, release

## Abstract

Biomedical cotton gauzes (**C0**), after a first functionalization with glycidyl methacrylate (GMA) by a Fenton’s reaction (material **C1**), can be further modified in order to make them suitable for the adsorption and next release of drugs. Indeed, either after opening the epoxide ring through the addition of water (material **C2**) or after the introduction of amino groups through reaction with diamines (1,2-diaminoethane (material **C3**), 1,6-diaminohexane (material **C4**) and 1,12-diaminododecane (material **C5**)), the new gauzes can be uploaded with drugs. Both ibuprofen (IB), a non-steroidal anti-inflammatory, and amoxicillin (AM), a wide-spectrum β-lactam antibiotic, are efficiently adsorbed from their aqueous solutions at 20 °C onto **C2**–**C5** (up to ≈0.8 mmol g^−1^ for IB and up to 0.4 mmol g^−1^ for AM) but not onto **C0** and **C1**. The release of both IB and AM is affected by the ionic strength of the medium in which the release takes place. Indeed, kinetic experiments conducted with a physiological solution (NaCl (aq, 0.9% *w*/*v*) showed good release efficiencies while only modest or negligible release was observed if deionised water was the release medium. Moreover, the kind of functionalization plays an important role during both the adsorption and the release. The gauzes **C3**–**C5** can be uploaded with a higher amount of drug relative to **C2**. Conversely, the drug is released quickly and in a higher amount from **C2** relative to the gauzes containing the amino groups.

## 1. Introduction

The management of the medical textiles in hospitals and medical centres is important for the health of the patients and for the safety of the medical and paramedical staff and has relevant economic implications [1,2,3]. The use of textiles and bioactive polymers with antimicrobial properties is often required in the case of people dramatically damaged by the fire or patients having decubitus ulcerations or suffering of diabetes mellitus [4,5,6,7,8,9]. Moreover, it is well known that textiles in clinical settings are excellent substrates for the growth of bacteria and fungi under appropriate moisture and temperature conditions [10]. In all these and analogous situations, the adoption of textiles with an antimicrobial capability is highly requested. In a more general view, textiles able to adsorb and release antibiotics [11,12], anti-inflammatories [13,14], antifungals [15], and any type of medicament or simply able to release perfumes and fragrances for healthcare [16], insect repellents [17], and insecticides [18] have huge marketing perspectives.

Cellulose-based textiles—in particular, those made with cotton, flax, hemp, ramie, or jute—are extensively used in many sectors of daily human life, especially in healthcare [19,20]. Modification of the cellulose fibres paves the way for almost infinite new applications, making them suitable for the manufacturing of functional and sustainable materials. The preparation of special cellulose fibres and textiles for medicine and healthcare has been attempted with different approaches and materials that include the use of silver nanoparticles [21], the surface functionalization with sol–gel treatments for the release of antioxidants [22], coating with silica nanoparticles functionalised with drugs [14,23], the incorporation of bio metal-organic framework [24] and coating with polylactic acid for the release of amoxicillin [25] and ibuprofen [13]. Moreover, chitosan, a biopolymer widely used for wound dressing applications due to its moderate antimicrobial activity against a wide spectrum of microorganisms, was also used for the coating of cotton fabrics [26,27,28,29]. Cellulose fibres functionalised with cyclodextrins have been also proposed over the years [30], allowing the adsorption and release of antifungals [31,32], antimicrobial agents [32,33,34,35], and insect repellents [36] due to the well-known ability of these oligosaccharides to create host–guest inclusion phenomena.

**Figure 1:**
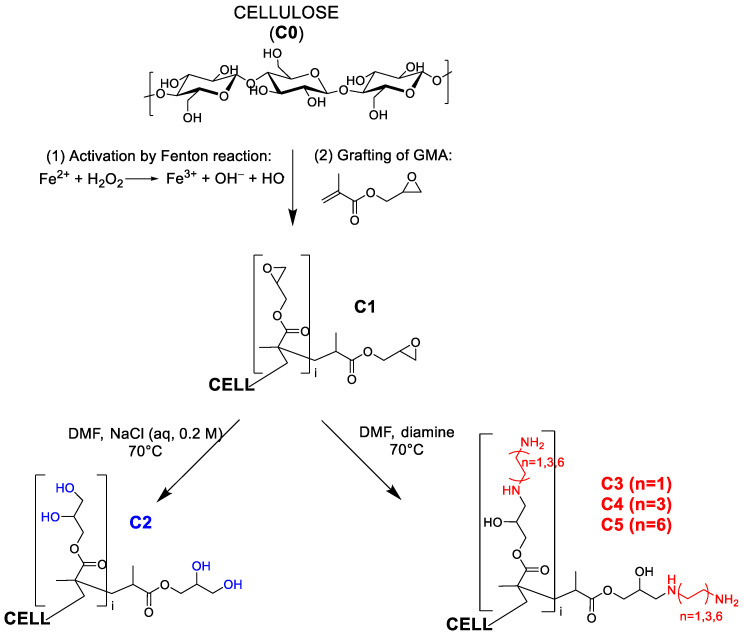
Scheme describing the preparation of the materials.

In this paper, cotton gauzes (**C0**) are covalently functionalised with glycidyl methacrylate (GMA) by Fenton reactions with different grafting levels (gauzes **C1**, see the scheme reported in Figure 1). These materials are then further modified by different chemical treatments. The gauzes **C2** are obtained after opening the epoxide ring of the grafted GMA by the addition of water while the gauzes **C3**–**C5** are prepared by reacting **C1** with linear diamines of different molecular weights. The adsorption and next release properties of these materials are investigated with regard to ibuprofen (IB) and amoxicillin (AM) as model molecules.

## 2. Results and Discussion

### 2.1. Preparation and Characterization of the Functionalised Cotton Gauzes

The grafting of different monomers (for example the derivatives of the acrylic and the methacrylic acid) onto cellulose can be promoted by redox processes based on free radicals that include, by the others, the use of Fenton’s reagents, cerium salts and persulphates [37,38,39,40]. Physical techniques based on high energy electron beams or plasma treatments can also be used with similar results. They have the advantage to be suitable for an industrial scale-up of the process [41,42,43]. The Fenton reaction does not require expensive reagents and equipment and can be easily carried out in academic research laboratories. It is synthetically represented by the following reaction:

Fe^2+^(aq) + H_2_O_2_(aq) → Fe^3+^(aq) + OH^−^(aq) + HO∙(aq)

Hydrogen peroxide is reductively degraded by means of Fe(II) ions in aqueous solution. This reaction leads to the formation of highly reactive and not-selective hydroxyl radicals that are able to activate the cellulose substrate by the formation of carbon radicals. Once formed, carbon radicals may be quenched by electrophilic molecules, such as those containing vinyl groups. GMA is a valuable monomer due to the presence of a terminal olefinic group able to react with carbon radicals generated on cellulose fibres and an epoxide group that could be used for further functionalization due to its reactivity toward nucleophilic reagents [44,45]. Moreover, polymers derived from GMA or other acrylic and methacrylic monomers have been tested for different biomedical applications [46,47,48].

The amount of GMA grafted on the cotton gauze can be modulated by changing the volume of GMA added during the reaction. **C1** samples having different moles of epoxide per gram of solid (in the following indicated as DG, degree of grafting, and expressed in mmol g^−1^) were prepared as described in the experimental section. The quantification of DG was obtained by elemental analysis after reacting **C1** with an excess of sodium azide (see Table 1 and Equation (1)) under the hypothesis that all epoxide groups react with the azide ions. This reaction is, indeed, very efficient due to the strong nucleophilicity of the N_3_^−^ ions and their small size [49,50]. From now on, a letter L or H between parentheses will be added whenever it is necessary to distinguish the **C1** samples with a low or a high DG, respectively. Starting from **C1** further modifications can be obtained either by opening the epoxide ring with DMF/NaCl (aq, 0.2 M) in order to prepare the material **C2** [44] or by introducing amino groups via the reaction of **C1** with aliphatic diamines (materials **C3**, derived from 1,2-diaminoethane (DAE), materials **C4**, derived from 1,6-diaminohexane (DAH), and materials **C5**, derived from 1,12-diaminododecane (DAD)). Moreover, the letters L and H will be associated with the materials **C2**, **C3**, **C4,** and **C5** depending on the DG values of **C1** from which they have been prepared. Table 2 reports the elemental analysis data for the materials **C3**, **C4,** and **C5** from which DG(*), a hypothetical DG value that would be obtained if a single diamine molecule reacted with a single epoxide unit (see Equation (2)), was calculated. For all materials, the ratio DG(*)/DG is always lower than 1, with values comprised between 0.6 and 0.8. There could be two possible motivations for this. One is that not all the epoxide groups reacted with the diamine molecules (even if a large excess of diamine was used in all preparations). Some epoxides could be protected from the nucleophilic attack of the diamines by the matrix of grafted polymer. However, the ^13^C CP/MAS and FT-IR spectra (see in the following) do not show the presence of unreacted epoxides. A second, probably more reliable, motivation is that there is a fraction of the grafted diamines that is attached to two epoxide groups. Finally, from the data in Table 2, it is possible to obtain an estimate of the amine content (primary and secondary) in the materials **C3**, **C4,** and **C5** (see the last column in Table 2).

**Figure 2:**
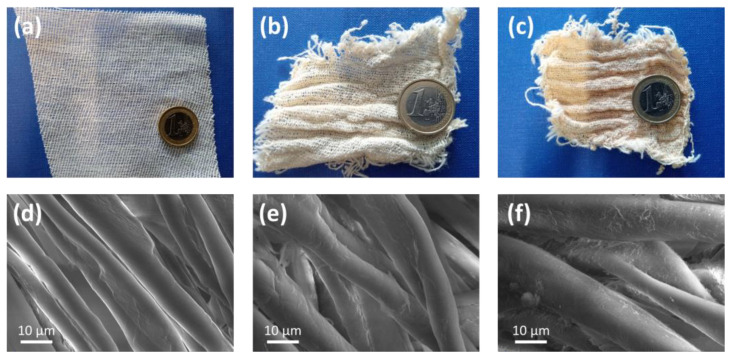
(**a**) Pristine cotton gauze (**C0**); (**b**) gauze **C1(H)**; (**c**) gauze **C4(H)**; SEM images of (**d**) **C0**; (**e**) **C1(L)**; (**f**) **C1(H)**.

Figure 2 shows the physical appearance of (a) the pristine cotton gauze, (b) the gauze **C1(H)** and (c) the gauze **C4(H)** as representative cases. The other materials have a quite similar aspect. The same figure also reports the SEM images of (d) **C0**, (e) **C1(L)**, and (f) **C1(H)**. Qualitatively speaking, the roughness of the cotton fibres surface was observed to increase progressively with DG due to the formation of grafted GMA polymeric layers. Indeed, the fibres of the pristine cotton appear to be quite smooth. However, no agglomeration of fibres is observed with the increasing of the DG, and the fibres are well separated and retain their original aspect.

**Figure 3:**
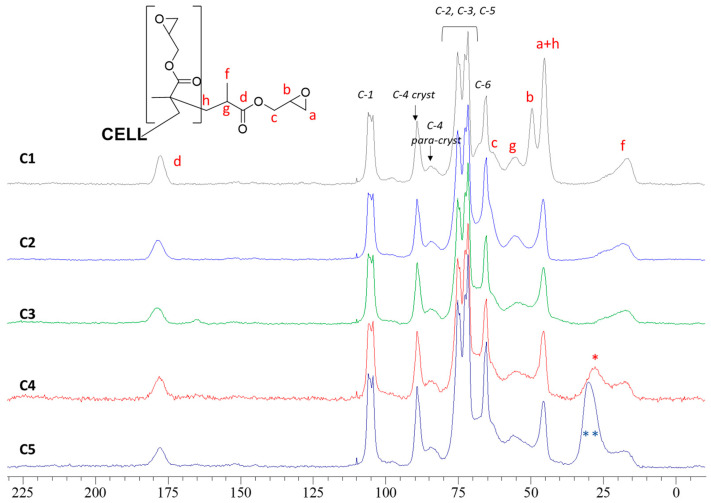
^13^C CP/MAS spectra of **C0**, **C1(H)**, **C2(H)**, **C3(H)**, **C4(H)** and **C5(H)**. Signals indicated with (*) and (**) are due to the methylene carbons of the diamines (excluding the alpha-carbons).

A further characterisation of the materials was done by ^13^C CP/MAS and FT-IR spectroscopies. Figure 3 shows the ^13^C CP/MAS spectra for **C1(H)** and **C2**–**C5** obtained from it. The grafting of GMA onto the cellulose polymer chains is confirmed by the appearance of new signals related to the GMA functional groups. In particular, it is possible to distinguish the signal of carbon of the ester group (~178 ppm), the signals of the epoxide (~45 ppm and ~42 ppm, this last one overlapping with the signal of -CH_2_ of GMA homopolymer), and the signal of the methyl group (~17 ppm). Finally, it is possible to clearly distinguish the signals of the carbons of cellulose. The functionalization procedures preserve the polymorphism of the native cellulose. Indeed, the signal of the carbon C-4 of the anhydroglucose unit is divided into two parts related to the crystalline and para-crystalline domains existing in the cellulose fibrils, and their ratio remains unmodified during all steps and is comparable to that of the pristine cotton gauzes (cryst:para-cryst ~63:37).

After the post-functionalisation of **C1** to obtain **C2**, **C3**, **C4**, and **C5**, the disappearance of the epoxide ring signals (in the range 40–50 ppm) is recognised. Instead, the >C=O (~178 ppm), the -CH_2_ of GMA homopolymer (~42 ppm), and the -CH_3_ (~17 ppm) signals of grafted GMA are preserved. With regard to **C3**, **C4**, and **C5**, the signal of the alpha-carbons of the grafted diamines unfortunately falls in the range 40–50 ppm and it is overlapped by the other signals. However, in **C4** and **C5**, the remaining methylene carbons of the diamines provide a signal in the range 25–35 ppm that is progressively more intense from **C4** to **C5**.

**Figure 4:**
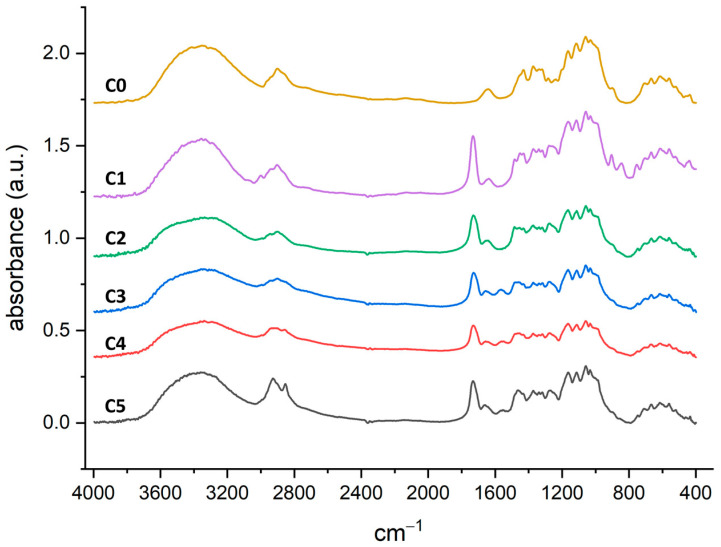
FTIR (KBr) spectra in absorbance mode.

The FT-IR spectra of all samples are reported in Figure 4 (the single spectra are also reported in the Supplementary Materials). In particular, in **C1** it is possible to detect the grafted-GMA signals at 3050–3000 cm^−1^ (weak, C–H epoxide ring stretching), 1730 cm^−1^ (strong, C=O ester stretching), 1270 cm^−1^, and 843 cm^−1^ (C–O–C epoxide ring stretching). The epoxide ring opening reaction to obtain **C2** as well as **C3**, **C4**, and **C5** causes the disappearance of the epoxide signals, while the GMA-carbonyl signal is preserved. Moreover, in **C3**, **C4**, and **C5**, it is possible to detect the -NH_2_ bending at 1560 cm^−1^ and, at 2850 cm^−1^, the C-H stretching of the grafted diamines. No modification of the spectra was observed after the treatment of the gauzes **C3**–**C5** with HCl 1.0 mM prior to the adsorption experiments (see the Supplementary Materials for a comparison between the spectra of **C4** before and after treatment with HCl 1.0 mM).

### 2.2. Adsorption and Release of Drugs

In order to study the applicability of the proposed materials as devices for the topical administration of drugs, AM and IB were used as test molecules. Amoxicillin (AM) is a wide-spectrum β-lactam antibiotic able to inhibits the bacterial cell wall synthesis. It is effective against a wide number of gram-positive and gram-negative bacteria, and it is administrated to patients in different formulations, either orally or topically. Ibuprofen (IB) is a non-steroidal anti-inflammatory drug that is used for the treatment of localised pain, arthritis, and musculoskeletal problems. Very preliminary indications that the material **C2** is able to adsorb and release AM were reported in two patents [51,52]. However, in reviewing the literature, it seems that there is a lack of systematic studies on the materials **C2**–**C5** and their interactions with drugs.

The adsorption kinetic measurements for IB and AM were performed at 20 °C. The initial concentration of IB solution was 5 mM, while for AM, a lower value (1.25 mM) was selected. The gauzes **C2(H)**–**C5(H)** were employed. As described in the experimental part, the materials **C3(H)**–**C5(H)** were washed with HCl (aq, 1 mM) in order to protonate the amines. Indeed, the presence of positive charges on the solid surface facilitates the adsorption of AM and, in particular, IB (in the form of sodium salt for solubility reasons).

**Figure 5:**
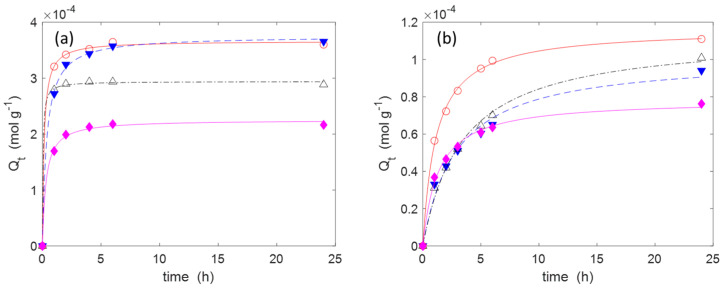
Adsorption kinetics of: (**a**) IB. (initial concentration: 5.0 mM); (**b**) AM (1.25 mM). Temperature: 20 °C. The markers indicate the experimental points, while the lines are obtained by a non-linear interpolation with a second-order kinetic model (Equation (4)). Legend: **C2(H)** (◆, ─), **C3(H)** (△, -∙-), **C4(H)** (○, ─), **C5(H)** (▼, --).

The experimental kinetic data (Figure 5) can be well-fitted with a pseudo-second-order (PSO) kinetic model (Equation (4)). A PSO rate equation is usually adopted for the analysis of the adsorption kinetics due its simple mathematical form. Further advantages and limitations are treated in the literature [53]. The parameters k_2_ and Q_e_, obtained through the non-linear fitting of the experimental data with a Matlab routine, are reported in Table 3 for both IB and AM. The adsorption of IB is quite fast, in particular on materials **C3**–**C5**. The equilibrium is, indeed, reached in few hours. The adsorption of AM is a bit slower. The material **C2** has a lower adsorption capacity toward both IB and AM compared to the other materials. Similar experiments conducted with the materials **C0** and **C1** did not show any adsorption of IB or AM.

(4) $$\frac{dQ_{t}}{dt}=k_{2}\cdot {\left(Q_{e}-Q_{t}\right)}^{2}$$

**Figure 6:**
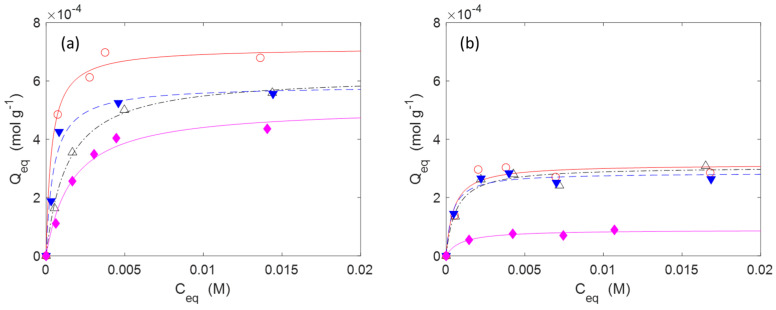
Adsorption isotherms of IB on: (**a**) materials with a high DG (**H**); (**b**) materials with a low DG (**L**). The markers indicate the experimental points while the lines are obtained by a non-linear interpolation with a Langmuir model (Equation (5)). Temperature: 20 °C. Legend: **C2** (◆, ─), **C3** (△, -∙-), **C4** (○, ─), **C5** (▼, --).

**Figure 7:**
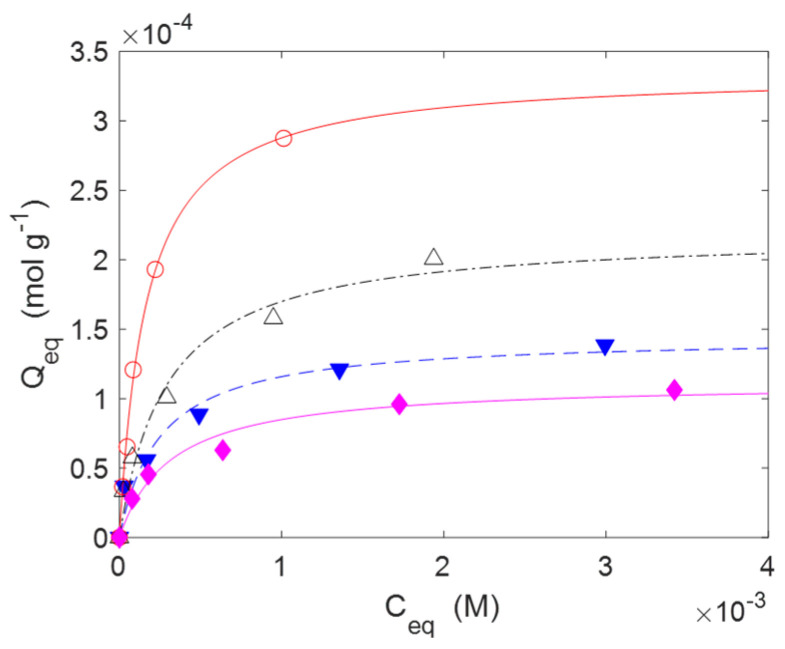
Adsorption isotherms of AM on materials with a high DG (**H**). The markers indicate the experimental points while the lines are obtained by a non-linear interpolation with a Langmuir model (Equation (5)). Temperature: 20 °C. Legend: **C2** (◆, ─), **C3** (△, -∙-), **C4** (○, ─), **C5** (▼, --).

The adsorption isotherms are reported in Figure 6 for IB and in Figure 7 for AM. The experiments were performed at 20 °C with contact times of 24 h long enough to reach the equilibrium. The adsorptions were carried out without using a buffer solution to fix the pH. However, the pH was monitored at the beginning of the adsorption and at the end. The pH values were quite close to neutrality in all cases. The experimental data were correlated through non-linear regression analysis with a Matlab routine using a Langmuir isotherm model, as described by the Equation (5). The model parameters are reported in Table 4. In Equation (5), *Q_m_* (mol g^−1^) is the maximum amount of drug that can be adsorbed onto the substrate at the specific temperature of the experiments (20 °C in this case), while *b* (M^−1^) is a constant related to the free energy of adsorption. *C_eq_* (M) is the concentration of the drug in the aqueous phase, while *Q_eq_* (mol g^−1^) is the amount of drug adsorbed onto the substrate in equilibrium conditions. Despite this model being based on hypotheses that often are not strictly fulfilled in real situations (like as the monolayer adsorption hypothesis), it is largely used for the analysis of reversible adsorption problems thanks to its simplicity and its capability to capture the key aspects of the molecular interactions at interfaces [54,55,56]. The reversibility condition is essential for materials designed for the release of drugs.

(5) $$Q_{eq}=\frac{Q_{m}\cdot b\cdot C_{eq}}{1+b\cdot C_{eq}}$$

In the case of IB, the data indicate that **C2** has a lower capability to adsorb this drug than **C3**–**C5** at any DG (H or L). This could be ascribed to the absence, in **C2**, of charged groups. At high DG, **C4** seems to have a better affinity for IB than **C3** and **C5** with higher *Q_m_* (7.144 × 10^−4^ mol g^−1^) and *b* (2.916 × 10^3^ M^−1^) values (Table 4). At low DG, there are no relevant differences between **C3**, **C4**, and **C5**.

The gauzes **C4(H)** also had a superior adsorption capacity with AM (Q_m_ = 3.681 × 10^−4^ mol g^−1^), while **C2(H)** was less efficient (Q_m_ = 1.15 × 10^−4^ mol g^−1^). In the case of AM, no experiments were carried out with materials with low DG. The range of concentrations of AM is limited by its solubility in water (≈5 mM at 20 °C, see Ref. [57] for more details).

Overall, the adsorption performances toward IB and AM not only depend on the presence of surface charges but also on the length of the grafted amines. Increasing this length could be beneficial for the adsorption because the drug molecules, according to their size, may find better accommodation between the molecular chains. However, long linear chains, as in the case of DAD in **C5**, are more hydrophobic and this hinders the adsorption of polar molecules like IB and AM. The higher hydrophobicity of **C5** relative to **C3** and **C4** was observed qualitatively during the time instants after the gauzes were immersed into the aqueous solutions containing the drugs. Indeed, for a few minutes, the **C5** samples tended to float on the solution, while **C3** and **C4** did not.

**Figure 8:**
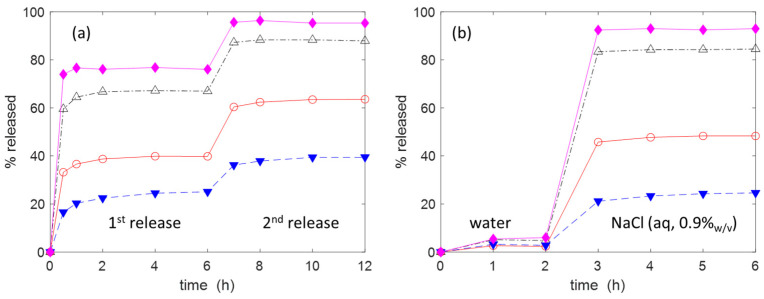
Cumulative release of IB in: (**a**) NaCl (aq, 0.9% *w*/*v*), initial quantities of IB loaded on the gauzes: **C2** (1.11 × 10^−4^ mol); **C3** (1.03 × 10^−4^ mol), **C4** (1.34 × 10^−4^ mol), **C5** (1.08 × 10^−4^ mol); (**b**) deionised water for the first 2 h and NaCl (aq, 0.9% *w*/*v*) for the remaining time, initial quantities of IB loaded on the gauzes: **C2** (6.673 × 10^−5^ mol); **C3** (6.668 × 10^−5^ mol), **C4** (8.536 × 10^−5^ mol), **C5** (8.361 × 10^−5^ mol). Temperature: 20 °C. Legend: **C2** (◆, ─), **C3** (△, -∙-), **C4** (○, ─), **C5** (▼, --).

**Figure 9:**
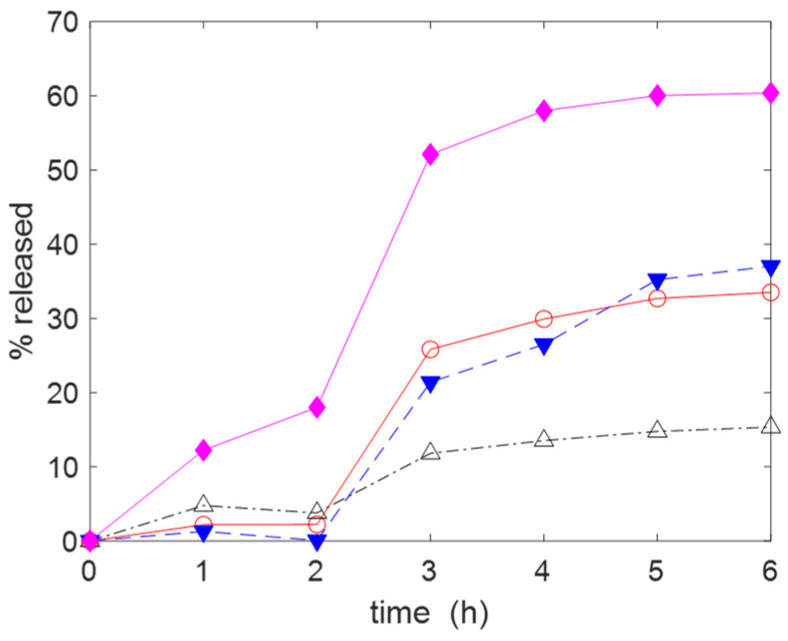
(Cumulative release of AM in deionised water for the first 2 h and NaCl (aq, 0.9% *w*/*v*) for the remaining time. Initial quantities of AM loaded on the gauzes: **C2** (1.652 × 10^−5^ mol), **C3** (2.343 × 10^−5^ mol), **C4** (2.757 × 10^−5^ mol), **C5** (1.945 × 10^−5^ mol). Temperature: 20 °C. Legend: **C2** (◆, ─), **C3** (△, -∙-), **C4** (○, ─), **C5** (▼, --).

The release of the drugs was carried out at 20 °C in 15 mL of a physiological solution (NaCl (aq, 0.9% *w*/*v*)) as this is often used for wound cleansing [58]. The gauzes **C2(H)**–**C5(H)** used for the release of IB were previously kept in contact with a 5 mM IB solution The initial amount of IB uploaded on each piece of gauze subjected to release was about 0.1 mmol (see the caption of Figure 8 for the details). The percentage of IB released was monitored over time. As shown in Figure 8a, the release curves trend toward their equilibrium value, which is reached in about 2 h. An interesting regularity in the release behaviour can be observed, with **C2** being the material with the higher release (≈76%), **C5** the material with the lower one (≈25%) and **C2** > **C3** > **C4** > **C5**. Due to the incomplete release, after 6 h, the aqueous phase was replaced with an equal volume of fresh saline solution. Doing so, it was possible to promote the release of a further amount of IB. The importance of the ionic strength of the release medium is made evident in the Figure 8b in which the release is carried out in 15 mL of deionised water for the first 2 h. A very low release was observed with all materials. After 2 h, the gauzes were transferred into 15 mL of saline solution, which caused the release of the adsorbed IB. Similar results were observed for the release of AM (see Figure 9). Therefore, the release of IB is strongly affected by the ionic exchange with the aqueous phase. Some simple calculations using the adsorption and release data and focusing the attention on AM provide a further support to the applicative potentiality of the proposed materials. Let us consider a wound with a surface area equal to 5 cm × 5 cm and a depth of 5 mm (so the wound volume is 12.5 mL). A depth of 5 mm can be considered a typical value for a large variety of wounds [4]. If a gauze with a weight of ≈0.8 g (and uploaded with AM, ≈0.2 mmol g^−1^) were applied on the wound and if only 10% of AM were released, then the concentration of antibiotic into the wound would be equal to ≈0.47 mg mL^−1^. This value is much higher than the minimum inhibitory concentration (MIC) of AM (MIC: 0.12–0.25 µg mL^−1^ for *S. aureus* strains [59]). MIC is the lowest concentration of drug that prevents visible in vitro growth of bacteria or fungi.

Finally, there is a further application field for the proposed materials that is out of the strictly biomedical field, although closely related to it. Currently, there is a high use of drugs for both human and veterinary pathologies. Considerable amounts of these molecules taken during the treatment of the pathologies are not retained by the body and are mainly excreted as still-active compounds. For this reason, high concentrations of drugs, including IB and AM, have been found in wastewater treatment plants, causing several ecological risks [60,61] From this point of view, the preparation of adsorbent filters made with **C2**–**C5** could also find a valid application as a filtration system for the removal of AM or IB from wastewater.

## 3. Materials and Methods

Cotton gauzes for medical uses with a size of about 10 cm × 10 cm and a weight of about 0.5 g/gauze were purchased online and used without any preliminary treatment. The reagents and solvents like H_2_O_2_ (30% *w*/*w*), FeSO_4_ × 7H_2_O, NaCl, glycidyl methacrylate (GMA), 2-propanol, dimethylformamide (DMF), 1,2-diaminoethane (DAE), 1,6-diaminohexane (DAH), 1,12-diaminododecane (DAD), and acetone are all commercially available and were used without further purification. Ibuprofen sodium salt (IB, Mw: 228.29 g mol^−1^) was obtained from Sigma-Aldrich. Amoxicillin trihydrate (AM, Mw: 419.45 g mol^−1^)) was purchased from TCI.

### 3.1. Materials Preparation

The materials **C1** and **C2** with two levels of GMA grafting were prepared according to a procedure already reported in a previous paper [45]. For **C1**, 6.0 g of **C0** were swelled for half an hour into 330 mL of water at 80 °C in a 500 mL round-bottom flask. Then, 8 mL of FeSO_4_ heptahydrate 0.05 M in water was added, followed by the addition of 17 mL of H_2_O_2_ 30% (*v*/*v*). The gauzes were kept under gentle stirring with the help of a spatula for 25 min at 80 °C. Then, a variable amount of GMA (2 mL for L samples and 6 mL for H samples) was added in one portion and under mixing at 80 °C for 15 min. The material was removed from the reaction vessel, washed 2 times with 200 mL acetone, and washed under reflux of acetone for 6 h using a Soxhlet extractor. The material was finally dried in air at room temperature for 72 h and under vacuum for 2 h.

The material **C2** was obtained by putting 3 g of **C1** in 100 mL of DMF at 80 °C for 2 h. Then, 100 mL of NaCl (aq, 0.2 M) solution was added. The liquid phase was kept under gentle stirring with a magnetic bar for 24 h at 80 °C. At the end, the samples were removed from the solution and exhaustively washed with water (about 250 mL × 4 times) and finally with acetone (about 100 mL × 2 times). The material was dried in air and finally under vacuum for 2 h.

The materials **C3**, **C4**, and **C5** were obtained in the following way. The gauzes **C2** (≈2.0 g) were added to 60 mL of a solution of diamine (≈15.0 mmol) in DMF at 20 °C. After 30 min, the temperature was raised to 70 °C, and the system was kept at this temperature for 24 h. The gauzes were finally removed from the DMF solution, washed with MeOH (100 mL for 2 times) and water (several times over 24 h). The material was dried in air for 72 h and under vacuum for 3 h.

In order to determine the DG values, 195 mg of NaN_3_ (3.0 mmol) was dissolved into a solution of DMF (3 mL) and water (400 µL). About 130 mg of **C1** were added and the mixture was kept under stirring at 70 °C for 24 h. The solid was recovered and extensively washed with water. It was dried in air for 48 h and under vacuum for 3 h.

### 3.2. Characterization

Solid-state ^13^C cross-polarization magic angle spinning (CP/MAS) spectra were collected at 125.77 MHz on a 500 MHz Bruker (Billerica, MA, USA) BioSpin NMR Spectrometer Avance 500 operating in a static field of 11.7 T and equipped with a 4 mm MAS probe, spinning the sample at the magic angle at speeds up to 15 kHz. This—with the addition of high-power ^1^H decoupling capability—allows a decrease in or the eliminate off homo- and heteronuclear anisotropies. All the samples were prepared by packing them in Zirconia (ZrO_2_) rotors closed with Kel-F caps (50 μL internal volume), and the magic angle spinning (MAS) rate was optimised to 10 kHz. Cross-polarization (CP) spectra under Hartmann−Hahn conditions were recorded with a variable spin-lock sequence (ramp CP-MAS) and acquired with a relaxation delay of 4 s, a ^1^H π/2 pulse width of 3.2 μs, a spectral width of 240 ppm (30,300 Hz), and an optimised contact time of 1.5 ms. The chemical shifts were recorded relative to CH Adamantane signal, previously acquired and used as standard reference.

The FT-IR analysis of the powdered sample with infrared grade KBr was obtained with a Bruker Tensor 27 spectrometer.

The elemental analysis was obtained with a Costech (Valencia, CA, USA) ECS 4010 analyser.

Scanning electron microscopy (SEM) was carried out by a variable pressure instrument (SEM Cambridge Stereoscan 360, Watertown, MA, USA) at 100/120 Pa with a detector VPSE. The operating voltage was 20 kV with an electron beam current intensity of 150 pA. The focal distance was 8 mm. The specimens were used without any treatment.

### 3.3. Drug Adsorption and Release

Before the adsorption experiments, all gauzes **C3**–**C5** were washed with HCl (aq, 1 mM) (100 mL for each sample, for 1 h 4 times). Finally, the materials were pressed between two foils of blotting paper and dried in air for 72 h and under vacuum for 2 h. The gauzes **C2** were used without this treatment.

All the adsorption experiments were performed in batch conditions. Aqueous solutions of the two drugs at different concentrations (ranging from 0.4 × 10^−4^ M to 4.4 × 10^−3^ M for AM and from 2.0 × 10^−3^ M to 20 × 10^−3^ M for IB) were prepared. Then, 20 mL of the drug solutions and about 200 mg of gauze were introduced into a 50 mL Falcon^®^ tube. The tubes were shaken at 260 rpm at 20 °C. The concentration of the solutions was determined by UV–Vis measurements using a Jasco (Easton, MD, USA) V-630 BIO UV-spectrophotometer. The molar absorption coefficients of the drugs in deionised water were ε_AM_ = 1071 M^−1^cm^−1^ (at 272 nm) for AM and ε_IB_ = 312 M^−1^cm^−1^ (at 264 nm) for IB. No relevant differences were observed with the saline solution The adsorption capacity was evaluated using the following formula:

*Q* = (*C*_0_ − *C*) × *V*/*m* (6)

where *C*_0_ and *C* are, respectively, the initial and the final concentration (M); *V* is the volume of the solution (L); and *m* is the mass of adsorbent (g).

After 24 h, the gauzes were removed from the drug solution and were pressed between two foils of blotting paper in order to remove the excess liquid. They were introduced in a 50 mL Falcon^®^ tube with 15 mL of NaCl (aq, 0.9% *w*/*v*) and shaken at 260 rpm at 20 °C. The amount of drug released at different times was measured using UV–Vis spectrometry. The percentage of drug released was calculated with the following Equation (3):

% released = 100 × (amount of drug released/amount of drug adsorbed)(7)

After 6 h, the gauzes were removed from the liquid, pressed with blotting paper, and put in contact with a fresh NaCl (aq, 0.9% *w*/*v*) solution. The release was measured as before. The cumulative percentage of release was calculated by adding up the different contributions. A similar experiment was conducted using deionised water for the first 2 h and then NaCl (aq, 0.9% *w*/*v*) for the remaining time.

## 4. Conclusions

This study provides evidence that it is possible to make cotton gauzes capable of adsorbing and subsequently releasing drugs after simple chemical modifications. Specifically, both the gauzes **C2**, in which the glycidyl moieties have been converted into glycerol moieties, and the gauzes **C3**–**C4**, obtained from the reaction of **C1** (gauzes functionalised with GMA by a Fenton’s reaction) with linear diamines of different molecular weights can be uploaded with IB or AM. The amount of drug adsorbed on each material depends on both the degree of grafting and the type of functionalization. The gauzes functionalised with diamines have the highest upload capability (≈0.714 mmol g^−1^ for IB and ≈0.335 mmol g^−1^ for AM on **C4(H)**), while the gauzes **C2** have the lowest one (≈0.512 mmol g^−1^ for IB and 0.112 mmol g^−1^ for AM on **C2(H)**). The drug release is affected by the kind of functionalization and by the ionic strength of the release medium.

## Figures and Tables

**Table 1:** Elemental analysis data of **C1** samples after reaction with NaN_3_ and DG values.

	GMA/Cellulose Preparative Ratio (mL/g)	N (%)	C (%)	H (%)	DG ^†^ (mmol g^−1^)
**C1(L)**	2/6	2.44 ± 0.1	41.42 ± 0.5	8.98 ± 0.3	0.596
**C1(H)**	6/6	7.48 ± 0.2	43.41 ± 0.5	9.25 ± 0.3	1.929
^†^ DG = 1000 × (N(%)/100)/(42 − 43 × (N(%)/100)					(1)

**Table 2:** Elemental analysis data of **C3**, **C4,** and **C5** samples and DG(*) values.

	Diamine ^§^	N (%)	C (%)	H (%)	DG(*) ^‡^	Amine Content (mmol g^−1^) ^†^
**C3(L)**	DAE (60.10)	1.08 ± 0.05	41.30 ± 0.5	9.19 ± 0.3	0.395	0.77
**C4(L)**	DAH (116.20)	1.08 ± 0.05	41.08 ± 0.5	9.65 ± 0.3	0.404	0.77
**C5(L)**	DAD (200.36)	0.87 ± 0.05	42.72 ± 0.5	9.32 ± 0.3	0.331	0.62
**C3(H)**	DAE (60.10)	3.91 ± 0.1	43.49 ± 0.5	10.30 ± 0.3	1.524	2.79
**C4(H)**	DAH (116.20)	3.14 ± 0.1	46.23 ± 0.5	10.19 ± 0.3	1.289	2.24
**C5(H)**	DAD (200.36)	2.56 ± 0.1	46.99 ± 0.5	11.77 ± 0.3	1.119	1.83
^‡^ DG(*) represents the DG that would be obtained if a single diamine molecule, having a molecular weight Mw (g mol^−1^), reacted with a single epoxide unit.						
DG(*) = 1000 × (N(%)/100)/(28 − Mw × (N(%)/100))						(2)
^§^ The molecular weight (g mol^−1^) is reported between commas.						
^†^ Amine content (mmol g^−1^) = 1000 × (%N/100)/14						(3)

**Table 3:** Kinetic constants (second-order model) for the adsorption of IB and AM (T = 20 °C).

	Ibuprofen 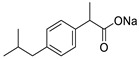		Amoxicillin 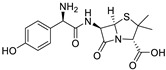	
	k_2_ (g mol^−1^ h^−1^)	Q_e_ (mol g^−1^)	k_2_ (g mol^−1^ h^−1^)	Q_e_ (mol g^−1^)
**C2(H)**	6.77 × 10^−5^	2.253 × 10^−4^	1.03 × 10^−4^	7.842 × 10^−5^
**C3(H)**	1.34 × 10^−5^	2.941 × 10^−4^	3.86 × 10^−4^	1.130 × 10^−4^
**C4(H)**	5.15 × 10^−5^	3.664 × 10^−4^	1.32 × 10^−4^	1.165 × 10^−4^
**C5(H)**	1.35 × 10^−5^	3.754 × 10^−4^	2.90 × 10^−4^	1.013 × 10^−4^

**Table 4:** Langmuir constants for the adsorption of IB and AM at 20 °C.

	IB		AM	
	Q_m_ (mol g^−1^)	b (M^−1^)	Q_m_ (mol g^−1^)	b (M^−1^)
**C2(L)**	8.942 × 10^−5^	1.066 × 10^3^	n.a.	n.a.
**C3(L)**	3.056 × 10^−4^	1.631 × 10^3^	n.a.	n.a.
**C4(L)**	3.317 × 10^−4^	2.113 × 10^3^	n.a.	n.a.
**C5(L)**	2.848 × 10^−4^	2.613 × 10^3^	n.a.	n.a.
**C2(H)**	5.120 × 10^−4^	6.146 × 10^2^	1.118 × 10^−4^	3.149 × 10^3^
**C3(H)**	6.210 × 10^−4^	7.542 × 10^2^	2.194 × 10^−4^	3.439 × 10^3^
**C4(H)**	7.144 × 10^−4^	2.916 × 10^3^	3.349 × 10^−4^	6.102 × 10^3^
**C5(H)**	5.845 × 10^−4^	2.114 × 10^3^	1.447 × 10^−4^	4.008 × 10^3^

## Data Availability

The original contributions presented in this study are included in the article/Supplementary Material. Further inquiries can be directed to the corresponding author.

## Supplementary Materials

The following supporting information can be downloaded at: https://www.mdpi.com/article/10.3390/molecules30030552/s1: from Figure S1 to Figure S8, individual FT-IR spectra of different materials; Figure S9, ^13^C CP/MAS NMR spectrum of **C0**; derivation of the Equations (S1)–(S3).

## References

[1] Vaishya R., Agarwal A.K., Tiwari M., Vaish A., Vijay V., Nigam Y. (2018). Medical textiles in orthopedics: An overview. J. Clin. Orthop. Trauma.

[2] Tassw D.F., Birlie B., Mamaye T. (2024). Nanotechnologies past, present and future applications in enhancing functionality of medical textiles: A review. J. Text. Inst..

[3] Firmanda A., Mahardika M., Fahma F., Amelia D., Pratama A.W., Amalia N., Syafri E., El Achaby M. (2024). Cellulose-enriched ascorbic acid for wound dressing application: Future medical textile. J. Appl. Polym. Sci..

[4] Shai A., Maibach H.I. (2005). Wound Healing and Ulcers of the Skin. Diagnosis and Therapy—The Practical Approach.

[5] Stoica A.E., Chircov C., Grumezescu A.M. (2020). Hydrogel Dressings for the Treatment of Burn Wounds: An Up-To-Date Overview. Materials.

[6] Frykberg R.G., Zgonis T., Armstrong D.G., Driver V.R., Giurini J.M., Kravitz S.R., Landsman A.S., Lavery L.A., Moore J.C., Schuberth J.M. (2006). Diabetic Foot Disorders: A Clinical Practice Guideline. J. Foot Ankle Surg..

[7] Atanasova D., Staneva D., Grabchev I. (2021). Textile Materials Modified with Stimuli-Responsive Drug Carrier for Skin Topical and Transdermal Delivery. Materials.

[8] Ongarora B.G. (2022). Recent technological advances in the management of chronic wounds: A literature review. Health Sci. Rep..

[9] Ahmadian Z., Adiban H., Rashidipour M., Eskandari M.R. (2022). Bioactive Natural and Synthetic Polymers for Wound Repair. Macromol. Res..

[10] Borkow G., Gabbay J. (2008). Biocidal textiles can help fight nosocomial infections. Med. Hypotheses.

[11] Rehan M., Zaghloul S., Mahmoud F.A., Montaser A.S., Hebeish A. (2017). Design of multi-functional cotton gauze with antimicrobial and drug delivery properties. Mater. Sci. Eng. C.

[12] Granados A., Pleixats R., Vallribera A. (2021). Recent Advances on Antimicrobial and Anti-Inflammatory Cotton Fabrics Containing Nanostructures. Molecules.

[13] Kopańska A., Brzeziński M., Gonciarz W., Draczyński Z. (2024). Compatibilizing of cotton fabric with hydrophobic drug cover layer for anti-inflammatory performance with the implementation of ibuprofen. Sci. Rep..

[14] Liu M., Guinart A., Granados A., Gimbert-Suriñach C., Fernández E., Pleixats R., Vallribera A. (2024). Coated Cotton Fabrics with Antibacterial and Anti-Inflammatory Silica Nanoparticles for Improving Wound Healing. ACS Appl. Mater. Interfaces.

[15] de Oliveira C.S.F., Tavaria F.K. (2023). The impact of bioactive textiles on human skin microbiota. Eur. J. Pharm. Biopharm..

[16] Martel B., Morcellet M., Ruffin D., Vinet F., Weltrowski L. (2002). Capture and Controlled Release of Fragrances by CD Finished Textiles. J. Incl. Phenom. Macrocycl. Chem..

[17] Hebeish A., Fouda M.M.G., Hamdy I.A., El-Sawy S.M., Abdel-Mohdy F.A. (2008). Preparation of durable insect repellent cotton fabric: Limonene as insecticide. Carbohydr. Polym..

[18] Hebeish A., El-Sawy S.M., Ragaei M., Hamdy I.A., El-Bisi M.K., Abdel-Mohdy F.A. (2014). New textiles of biocidal activity by introduce insecticide in cotton-poly (GMA) copolymer containing β-CD. Carbohydr. Polym..

[19] Kvavadze E., Bar-Yosef O., Belfer-Cohen A., Boaretto E., Jakeli N., Matskevich Z., Meshveliani T. (2009). 30,000-Year-Old Wild Flax Fibres. Science.

[20] Shahriari-Khalaji M., Alassod A., Nozhat Z. (2022). Cotton-based health care textile: A mini review. Polym. Bull..

[21] Zhang D., Chen L., Zang C., Chen Y., Lin H. (2013). Antibacterial cotton fabric grafted with silver nanoparticles and its excellent laundering durability. Carbohydr. Polym..

[22] Puoci F., Saturnino C., Trovato V., Iacopetta D., Piperopoulos E., Triolo C., Bonomo M.G., Drommi D., Parisi O.I., Milone C. (2020). Sol–Gel Treatment of Textiles for the Entrapping of an Antioxidant/Anti-Inflammatory Molecule: Functional Coating Morphological Characterization and Drug Release Evaluation. Appl. Sci..

[23] Li H., Granados A., Fernández E., Pleixats R., Vallribera A. (2020). Anti-inflammatory Cotton Fabrics and Silica Nanoparticles with Potential Topical Medical Applications. ACS Appl. Mater. Interfaces.

[24] Noorian S.A., Hemmatinejad N., Navarro J.A.R. (2019). BioMOF@cellulose fabric composites for bioactive molecule delivery. J. Inorg. Biochem..

[25] Macha I.J., Muna M.M., Magere J.L. (2018). In vitro study and characterization of cotton fabric PLA composite as a slow antibiotic delivery device for biomedical applications. J. Drug Deliv. Technol..

[26] Zhang Z., Chen L., Ji J. (2003). Antibacterial Properties of Cotton Fabrics Treated with Chitosan. Text. Res. J..

[27] Lim S.H., Hudson S.M. (2004). Application of a fiber-reactive chitosan derivative to cotton fabric as an antimicrobial textile finish. Carbohydr. Polym..

[28] Gupta B., Arora A., Saxena S., Sarwar A.M. (2009). Preparation of chitosan–polyethylene glycol coated cotton membranes for wound dressings: Preparation and characterisation. Polym. Adv. Technol..

[29] Ruan H., Aulova A., Ghai V., Pandit S., Lovmar M., Mijakovic I., Kádár R. (2023). Polysaccharide-based antibacterial coating technologies. Acta Biomater..

[30] Radu C.D., Parteni O., Ochiuz L. (2016). Applications of cyclodextrins in medical textiles—Review. J. Control. Release.

[31] Wang J.H., Cai Z. (2008). Incorporation of the antibacterial agent, miconazole nitrate into a cellulosic fabric grafted with b-cyclodextrin. Carbohydr. Polym..

[32] Hedayati N., Montazer M., Mahmoudirad M., Toliyat T. (2020). Ketoconazole and Ketoconazole/β-cyclodextrin performance on cotton wound dressing as fungal skin treatment. Carbohydr. Polym..

[33] Qian L., Guan Y., Ziaee Z., He B., Zheng A., Xiao H. (2009). Rendering cellulose fibres antimicrobial using cationic, β-cyclodextrin-based polymers included with antibiotics. Cellulose.

[34] Hiriart-Ramírez E., Contreras-García A., Garcia-Fernandez M.J., Concheiro A., Alvarez-Lorenzo C., Bucio E. (2012). Radiation grafting of glycidyl methacrylate onto cotton gauzes for functionalization with cyclodextrins and elution of antimicrobial agents. Cellulose.

[35] Abdel-Halim E.S., Al-Deyab S.S., Alfaifi A.Y.A. (2014). Cotton fabric finished with -cyclodextrin: Inclusion ability toward antimicrobial agent. Carbohydr. Polym..

[36] Romi R., Lo Nostro P., Bocci E., Ridi F., Baglioni P. (2005). Bioengineering of a Cellulosic Fabric for Insecticide Delivery via Grafted Cyclodextrin. Biotechnol. Prog..

[37] Yu H., Fu G., He B. (2007). Preparation and adsorption properties of PAA-grafted cellulose adsorbent for low-density lipoprotein from human plasma. Cellulose.

[38] Roy D., Semsarilar M., Guthrie J.T., Perrier S. (2009). Cellulose modification by polymer grafting: A review. Chem. Soc. Rev..

[39] Hsu S.T., Chen L.C., Lee C.C., Pan T.C., You B.X., Yan Q.F. (2009). Preparation of methacrylic acid-modified rice husk improved by an experimental design and application for paraquat adsorption. J. Hazard. Mater..

[40] Lumbreras-Aguayo A., Meléndez-Ortiz H.I., Puente-Urbina B., Alvarado-Canché C., Ledezma A., Romero-García J., Betancourt-Galindo R. (2019). Poly(methacrylic acid)-modified medical cotton gauzes with antimicrobial and drug delivery properties for their use as wound dressings. Carbohydr. Polym..

[41] Andreozzi L., Castelvetro V., Ciardelli G., Corsi L., Faetti M., Fatarella E., Zulli F. (2005). Free radical generation upon plasma treatment of cotton fibers and their initiation efficiency in surface-graft polymerization. J. Colloid Interface Sci..

[42] Alberti A., Bertini S., Gastaldi G., Iannaccone N., Macciantelli D., Torri G., Vismara E. (2005). Electron beam irradiated textile cellulose fibres.: ESR studies and derivatisation with glycidyl methacrylate (GMA). Eur. Polym. J..

[43] Elmaaty T.A., Okubayashi S., Elsisi H., Abouelenin S. (2022). Electron beam irradiation treatment of textiles materials: A review. J. Polym. Res..

[44] Vismara E., Melone L., Gastaldi G., Cosentino C., Torri G. (2009). Surface functionalization of cotton cellulose with glycidyl methacrylate and its application for the adsorption of aromatic pollutants from wastewaters. J. Hazard. Mater..

[45] Vismara E., Melone L., Torri G., Giuliano B., Vinci E.J. (2012). Surface Functionalizationed Cotton with Glycidyl Methacrylate: Physico-Chemical Aspects and Multitasking Applications. Cotton: Cultivation, Varieties and Uses.

[46] Dawlee S., Jayakrishnan A., Jayabalan M. (2009). Studies on novel radiopaque methyl methacrylate: Glycidyl methacrylate based polymer for biomedical applications. J. Mater. Sci. Mater. Med..

[47] Dos Santos J.F.R., Torres-Labandeira J.J., Matthijs N., Coenye T., Concheiro A., Alvarez-Lorenzo C. (2010). Functionalization of acrylic hydrogels with α-, β- or γ-cyclodextrin modulates protein adsorption and antifungal delivery. Acta Biomater..

[48] Travan A., Marsich E., Donati I., Benincasa M., Giazzon M., Felisari L., Paoletti S. (2011). Silver–polysaccharide nanocomposite antimicrobial coatings for methacrylic thermosets. Acta Biomater..

[49] Tsarevsky N.V., Bencherif S.A., Matyjaszewski K. (2007). Graft Copolymers by a Combination of ATRP and Two Different Consecutive Click Reactions. Macromolecules.

[50] Zhang Q., Slavin S., Jones M.W., Haddleton A.J., Haddleton D.M. (2012). Terminal functional glycopolymers via a combination of catalytic chain transfer polymerisation (CCTP) followed by three consecutive click reactions. Polym. Chem..

[51] Vismara E., Melone L., Torri G., Graziani G., Montanelli A. (2016). Derivatised Polysaccharide Material for the Topic Antibacterial Activity. EP2182931 (B1).

[52] Vismara E., Melone L., Torri G., Graziani G., Montanelli A. (2010). Method and Kit for Antibiogram. WO2010/010582 A1.

[53] Bullen J.C., Saleesongsom S., Gallagher K., Weiss D.J. (2021). A Revised Pseudo-Second-Order Kinetic Model for Adsorption, Sensitive to Changes in Adsorbate and Adsorbent Concentrations. Langmuir.

[54] Swenson H., Stadie N.P. (2019). Langmuir’s Theory of Adsorption: A Centennial Review. Langmuir.

[55] Hamdaoui O., Naffrechoux E. (2007). Modeling of adsorption isotherms of phenol and chlorophenols onto granular activated carbon: Part I. Two-parameter models and equations allowing determination of thermodynamic parameters. J. Hazard. Mater..

[56] Hamdaoui O., Naffrechoux E. (2007). Modeling of adsorption isotherms of phenol and chlorophenols onto granular activated carbon: Part II. Models with more than two parameters. J. Hazard. Mater..

[57] Felix I.M.B., Moreira L.C., Chiavone-Filho O., Mattedi S. (2016). Solubility measurements of amoxicillin in mixtures of water and ethanol from 283.15 to 298.15 K. Fluid Phase Equilib..

[58] Fernandez R., Green H.L., Griffiths R., Atkinson R.A., Ellwood L.J. (2022). Water for wound cleansing. Cochrane Database Syst. Rev..

[59] Andrews J.M. (2001). Determination of minimum inhibitory concentrations. J. Antimicrob. Chemother..

[60] Putra E.K., Pranowo R., Sunarso J., Indraswati N., Ismadji S. (2009). Performance of activated carbon and bentonite for adsorption of amoxicillin from wastewater: Mechanisms, isotherms and kinetics. Water Res..

[61] Basha S., Barr C., Keane D., Nolan K., Morrissey A., Oelgemőllerd M., Tobin J.M. (2011). On the adsorption/photodegradation of amoxicillin in aqueous solutions by an integrated photocatalytic adsorbent (IPCA): Experimental studies and kinetics analysis. Photochem. Photobiol. Sci..

