# Supplementary material for: The Role of the Functionalization of Biomedical Fabrics on Their Ability to Adsorb and Release Drugs
Source: Molecules. 2025 Jan 25;30(3):552. doi: 10.3390/molecules30030552 (PMC11819996; doi:10.3390/molecules30030552)
Supplement: Supplementary file 1 [file molecules-30-00552-s001.zip › molecules-3421709-supplementary.pdf]

Supplementary Materials

to

The Role of the Functionalization of Biomedical Fabrics on Their Ability to Adsorb and Release Drugs

Lucio Melone [1,2]

[1] Department of Chemistry, Materials and Chemical Engineering "G.Natta", Politecnico di Milano, Via Mancinelli 7, 20131 Milano, Italy; lucio.melone@polimi.it

[2] Department of Theoretical and Applied Sciences, eCampus University, Via Isimbardi 10, 22060 Novedrate, Italy; lucio.melone@uniecampus.it

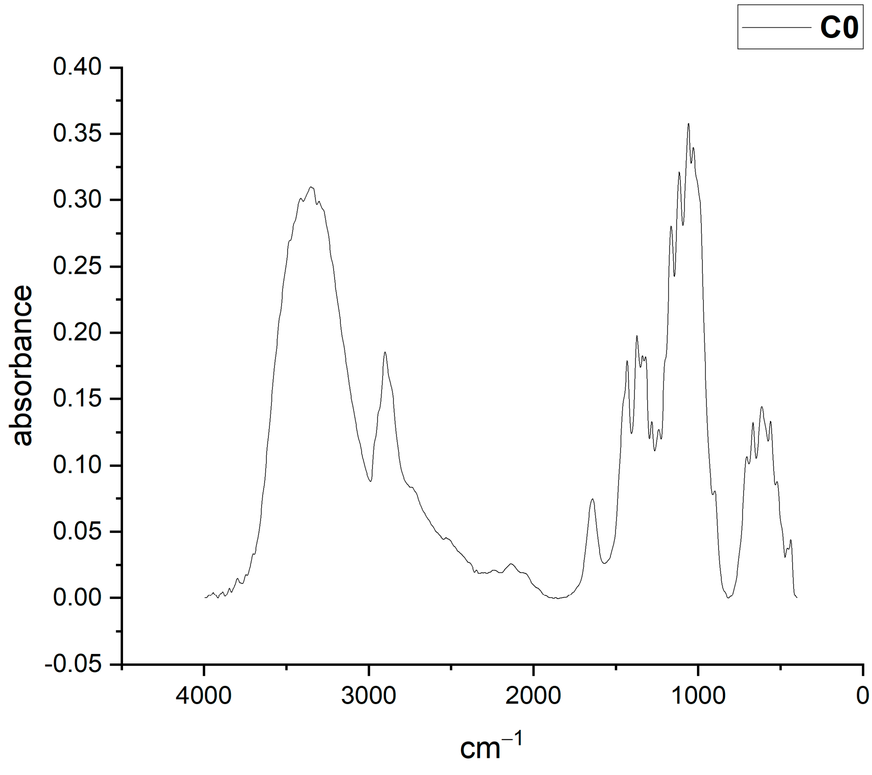

Figure S1. FT-IR spectrum of **C0** (KBr).

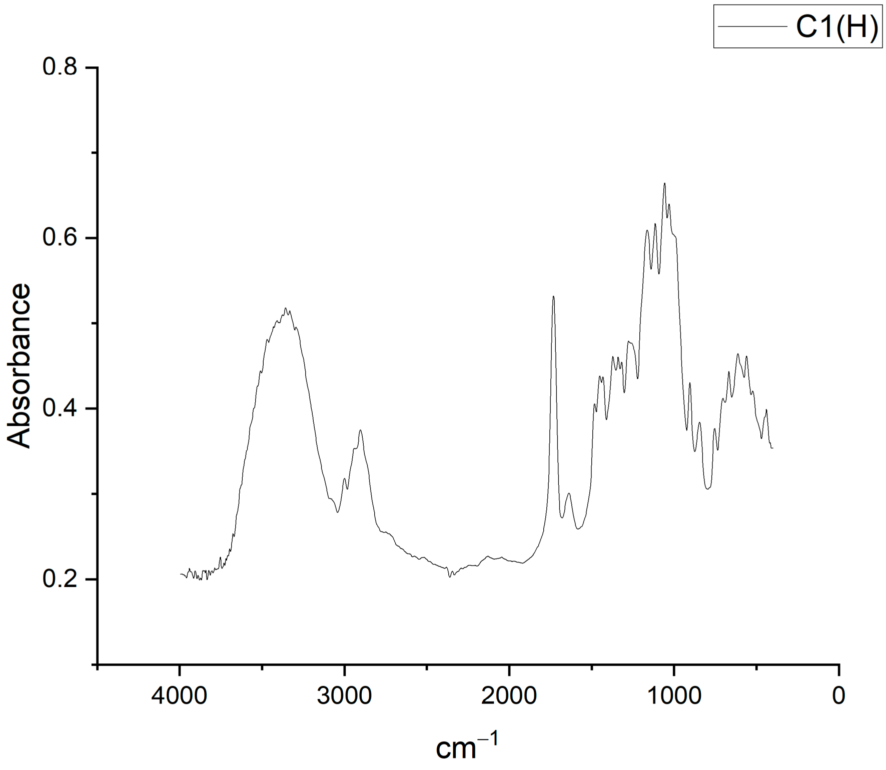

Figure S2. FT-IR spectrum of **C1(H)** (KBr).

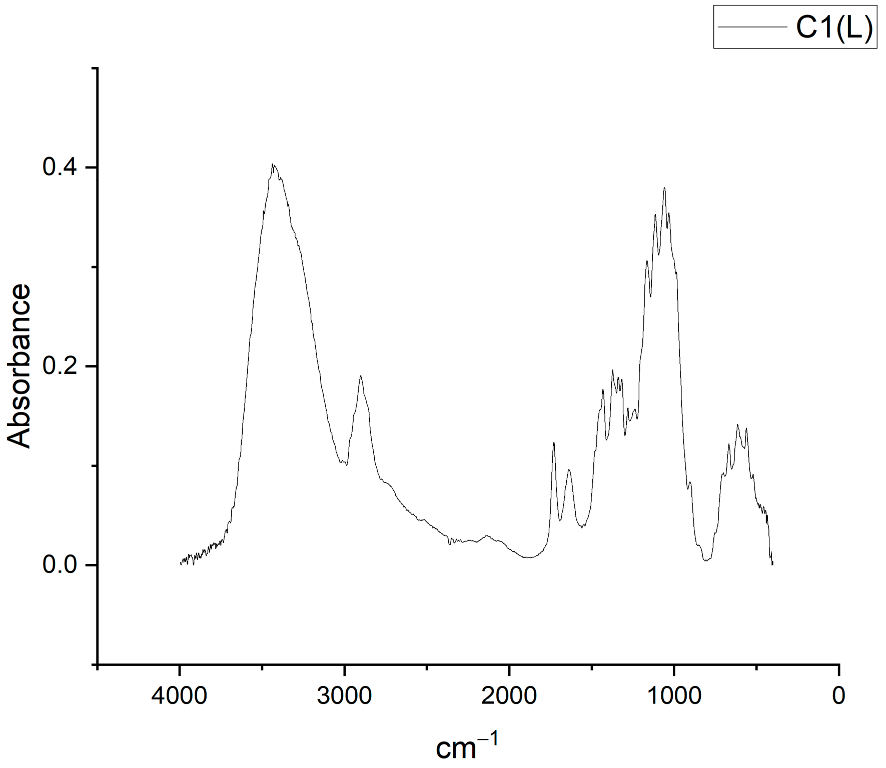

Figure S3. FT-IR spectrum of **C1(L)** (KBr).

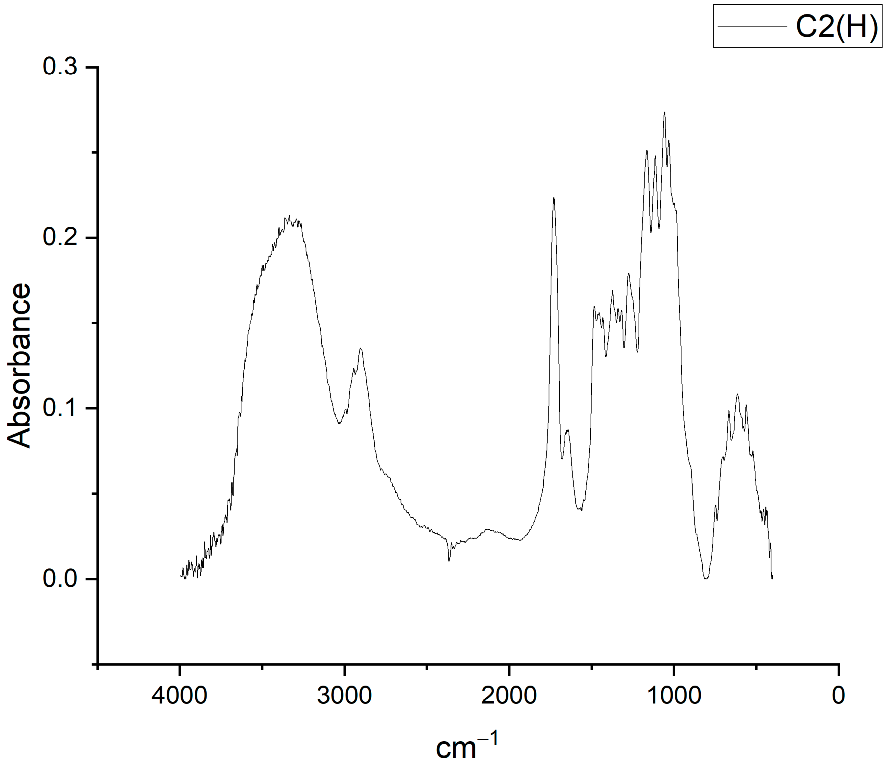

Figure S4. FT-IR spectrum of **C2(H)** (KBr).

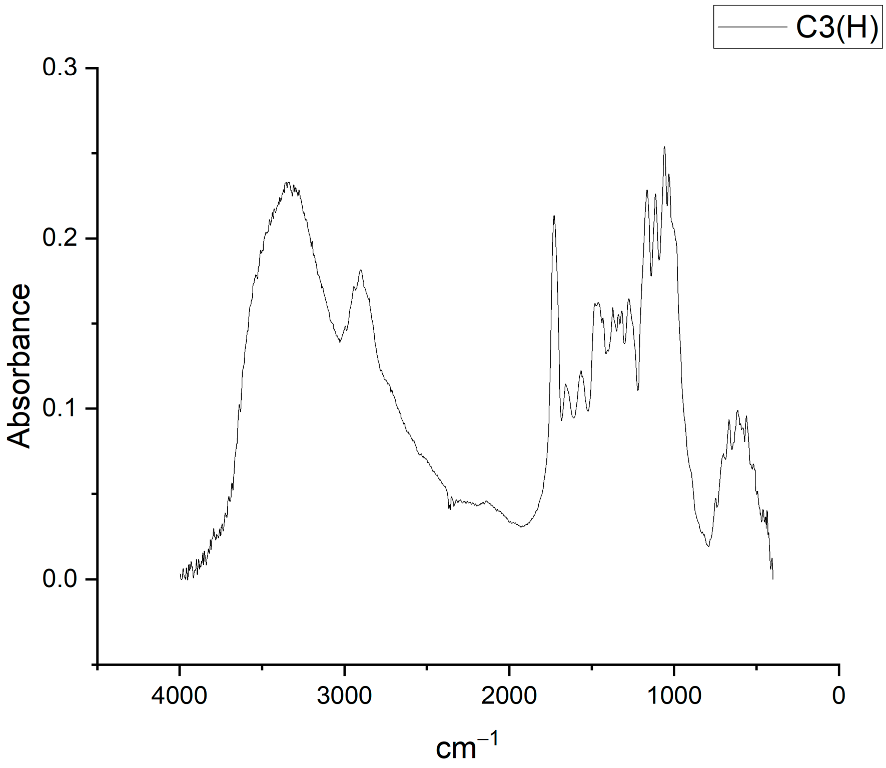

Figure S5. FT-IR spectrum of **C3(H)** (KBr).

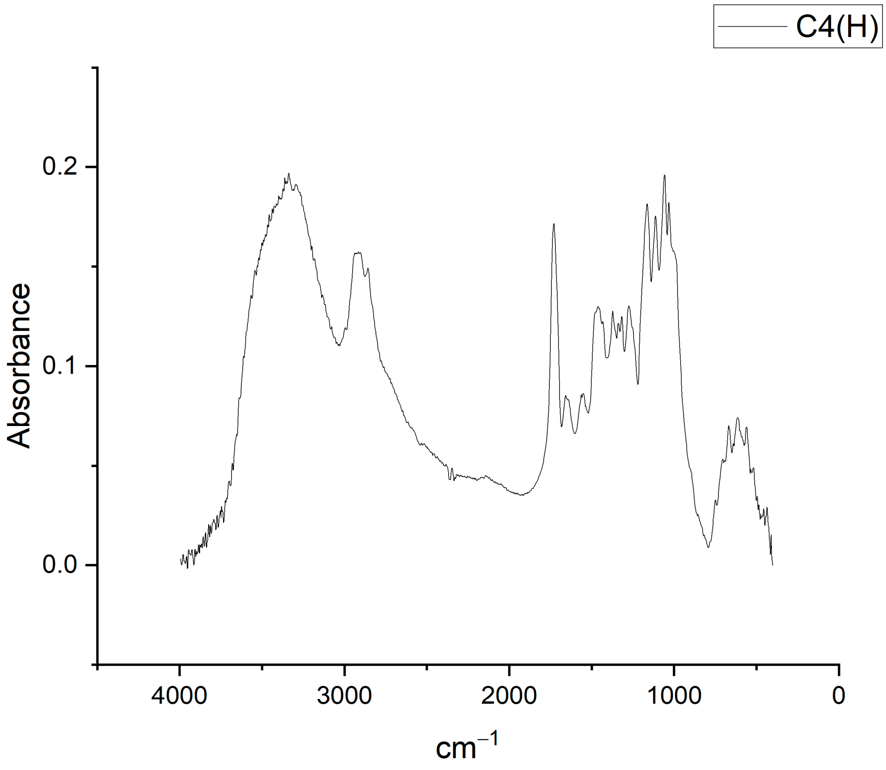

Figure S6. FT-IR spectrum of **C4(H)** (KBr).

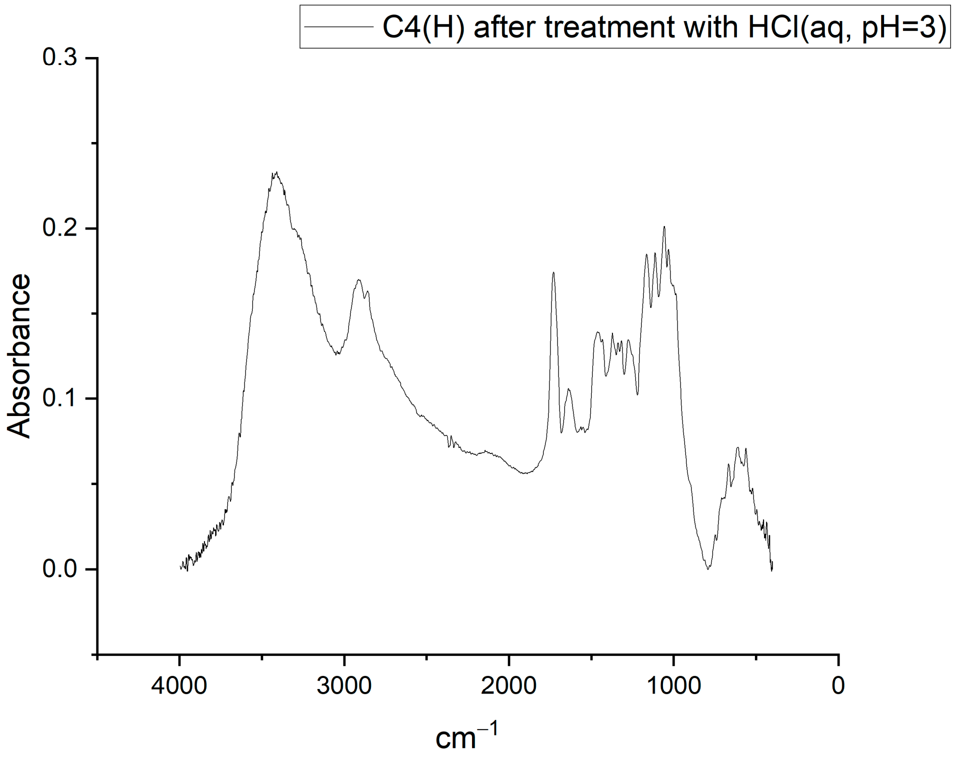

Figure S7. FT-IR spectrum of **C4(H)** after the treatment with HCl (1mM) (KBr).

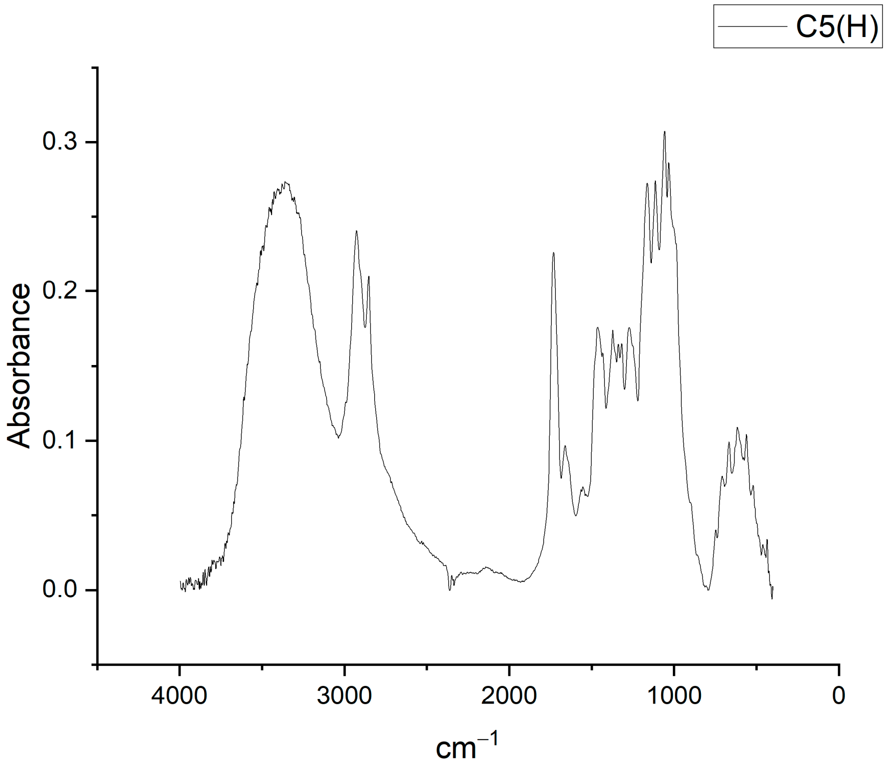

Figure S8. FT-IR spectrum of **C5(H)** (KBr).

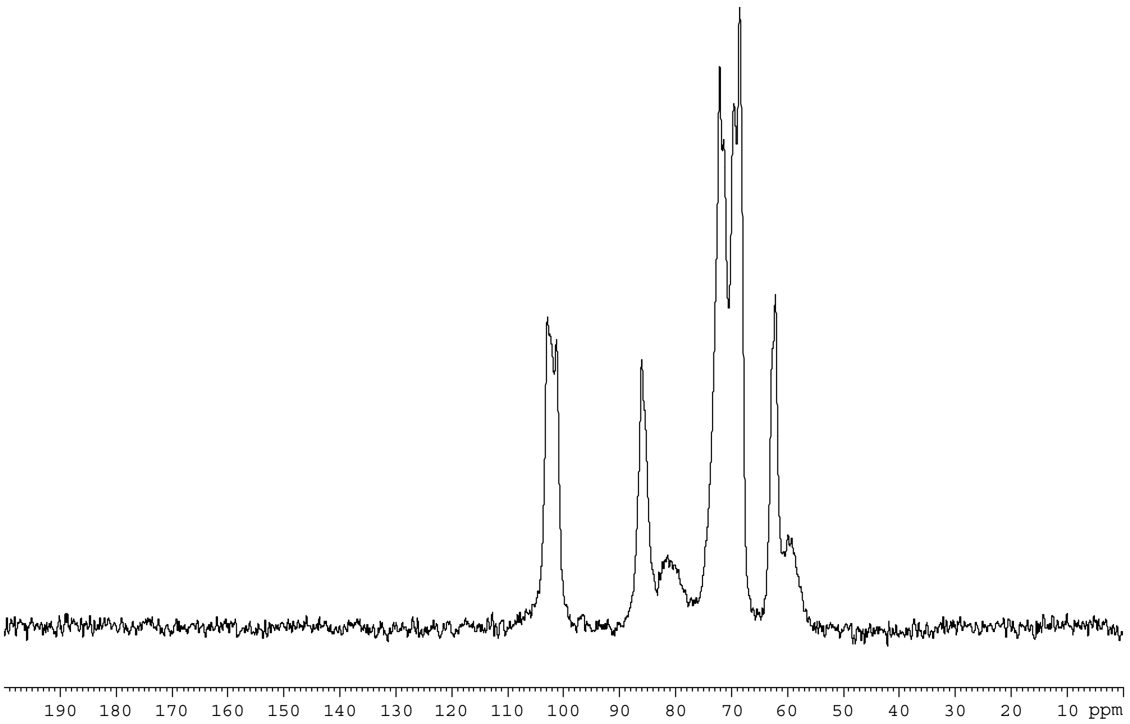

Figure S9. ^{13}C CP/MAS NMR spectrum of **C0**.

Equation S1

The degree of grafting is defined as:

$$DG = \frac{mmol\ epoxide}{g\ of\ \boldsymbol{C1}}$$

If %N is the mass percentage of nitrogen obtained from the elemental analysis of the sample **C1** after reaction with sodium azide, we can write:

$$\%N = \frac{g\ of\ N}{g\ of\ \boldsymbol{C1}(N_3)} \times 100 = \frac{DG \times (42/1000)}{1 + DG \times (43/1000)} \times 100$$

From this we can obtain the Eq.1 reported in Table 1:

$$DG = 1000 \times \frac{\left(\frac{\%N}{100}\right)}{\left(42 - 43 \times \frac{\%N}{100}\right)}$$

Equation S2

DG(*) (the hypothetical DG value that would be obtained if a single diamine molecule reacted with a single epoxide unit) is obtained in the following way:

$$\%N = \frac{g\ of\ N}{g\ of\ \boldsymbol{Ck}} \times 100 = \frac{DG^* \times (28/1000)}{1 + DG^* \times \left(\frac{MW}{1000}\right)} \times 100 \qquad k = 3, 4, 5$$

Then, DG(*) is given by:

$$DG^* = 1000 \times \frac{\left(\frac{\%N}{100}\right)}{\left(28 - MW \times \frac{\%N}{100}\right)}$$

Equation S3

The content of amines (primary and secondary amines) is simply:

$$Amine\ content\ (mmol\ g^{-1}) = 1000 \times \frac{(\%N/100)}{14}$$
